# Analysis of access to health services in Brazil according to sociodemographic profile: National Health Survey, 2019

**DOI:** 10.1590/S2237-96222022000300013

**Published:** 2022-12-19

**Authors:** Nathalia Campos Palmeira, Julia Pustrelo Moro, Fabiana de Abreu Getulino, Yohana Pereira Vieira, Abelardo de Oliveira Soares, Mirelle de Oliveira Saes

**Affiliations:** 1 Universidade Federal do Rio Grande, Faculdade de Medicina, Rio Grande, RS, Brazil; 2 Universidade Federal do Rio Grande, Programa de Pós-Graduação em Ciências da Saúde, Rio Grande, RS, Brazil

**Keywords:** Health Services, Access to Health Services, Equity in Access, Socioeconomic Factors, Epidemiological Surveys

## Abstract

**Objective:**

to describe the access to and utilization of health services among the Brazilian population according to sociodemographic characteristics, based on the 2019 National Health Survey (*Pesquisa Nacional de Saúde - PNS*).

**Methods:**

this was a cross-sectional descriptive study based on a PNS sample; the prevalence and respective confidence intervals of data stratified by sex, schooling, age and national macro-region of residence were calculated; data were analyzed using Stata software version 16.1.

**Results:**

a total of 293,725 individuals were interviewed; males showed lower proportion of medical consultations (66.6%) and were less likely to seek care (17.6%); among those living in the North region, 69.1% had medical consultations; 16.5% of individuals with low level of education obtained medication through the Brazilian Popular Pharmacy Program.

**Conclusion:**

the results reinforce iniquities in access to and utilization of health services, in addition to the need for monitoring indicators in order to guide health policies in Brazil.

## INTRODUCTION

The services offered by the Brazilian National Health System (*Sistema Único de Saúde -SUS*) range from actions aimed at preventing diseases and health problems to diagnosis, treatment and rehabilitation of affected people, in addition to health promotion and maintenance. In Brazil, universal and free access to these services is a right guaranteed by the 1988 Federal Constitution and by the SUS, which was created in 1990.^1,2^ However, access to those services depends on both supply and availability and on the perception and needs of the individual and community.^3^

In Brazil, population surveys have identified an increase in the supply of health actions and services, especially in the period from 2008 to 2013. However, it could be seen the maintenance of inequalities, with greater difficulty to access health services faced by users with low level of education, low-income population and residents in the North and Northeast regions of the country.^4–6^ It is common knowledge that inequality in access to services leads to the worst outcomes and health problems for the general population.^7^

National and international studies have emphasized that the increase in the supply of health services is not enough to ensure greater access and use of these services, especially among the most vulnerable populations.^8,9^ In recent years, the SUS has undergone important changes in its programs and policies, especially in Primary Health Care (PHC); among these changes, it is worth highlighting the new Brazilian National Primary Healthcare Policy, in 2017, and the Prevent Brazil Program, in 2019, which reformulated the primary health care financing policy.^10,11^ Since then, as a result of the new health policies, the estimate of PHC funding within the SUS has been based on the number of citizens registered in the municipalities and the performance achieved by health teams, the latter evaluated through indicators and goals defined by the Ministry of Health. In addition, new possibilities of relationship between the Government and private companies have come into force, in such a way that public and private sectors can participate, indistinctly, in the provision of health services within the SUS, resulting in the reduction of the state's duty, defined constitutionally, to promote health care for the Brazilian population. Thus, the expansion in access and service quality tends to be reduced, given that possible funding distortion may restrict the performance of PHC.^10,11^ In this sense, identifying the gaps in access to and utilization of public health services in this new scenario can contribute to identifying needs and reducing iniquities in universal access to services.

**Table:** 

Study contributions	
**Main results**	The use of dental services and health care visits aimed at seeking medicines, either through public services or through the Brazilian Popular Pharmacy Program, was more frequent for people living in the North and Northeast regions, and among individuals with low level of education.
**Implications for services**	To help health managers to guide health policies, according to the needs of each region, in addition to providing access and availability, and also make the services offered by the Brazilian National Health System more effective.
**Perspectives**	It is expected that health indicators of the Brazilian population will present a reduction in the iniquity in access to and use of health services.

The aim of this study was to describe the access to and utilization of health services by the Brazilian population according to sociodemographic characteristics, based on data from the 2019 National Health Survey (*Pesquisa Nacional de Saúde - PNS*).

## METHODS

This was a descriptive cross-sectional study, in which data from the 2019 PNS, a population-based household survey conducted by the Brazilian Institute of Geography and Statistics (*Instituto Brasileiro de Geografia e Estatística -IBGE*) in partnership with the Ministry of Health, were analyzed. This survey aims to produce data on the health situation and lifestyle of the Brazilian population, as well as on health care at the national level, with regard to access and utilization of services, preventive actions, continuity of care and health care financing.

The 2019 PNS sampling was estimated by clusters in three stages of selection. In the first stage, primary sampling units (PSUs) were stratified by municipalities, through simple random selection. The sample size was defined in 8,036 PSUs, which represent 53% of the PSUs and correspond to a set of area units from which it is possible to select subsamples that meet the survey objective. In the second stage, the distribution of households by PSU were carried out, where the interviews would be conducted: for Federative Units (FUs) with the largest number of PSUs, the smallest number of households (12 households); for FUs with the smallest number of PSUs, the largest number of households (18 households); and finally, for FUs that had not been included in the two previously defined criteria, the number established was 15 households per UPA. In the third stage, a resident aged 15 years and older was selected, with equiprobability among all adult residents of the household, to answer the individual interview. In this study, the additional eligibility criterion was to have answered questions about access to and utilization of SUS and private health services, regardless of sex and place of residence.

More detailed descriptions of the sampling process and data collection methods can be found in the 2019 PNS report, available in the IBGE website. Data were accessed on July 21, 2021.

PNS data collection took place between August 2019 and March 2020, by means of the proposition of a questionnaire comprised of three blocks of information: a) household variables; b) general characteristics of all residents of the household; and c) questions about work and health, directed to a randomly selected resident. The questionnaire was applied by properly trained interviewers, according to the personal digital assistant (PDA). The interviews were scheduled according to the availability of each resident, and two or more attempts to visit each household were established.

The studied variables that were related to the utilization of services, referred to the following questions:

- Stopped performing usual activities for health reasons in the last two weeks;
- Sought medical care from the same medical professional or sought for the same health service in the last two weeks;
- Had a doctor's appointment in the last 12 months;
- Had a dentist appointment in the last 12 months;
- Sought medical care in health services in the last two weeks;
- Got medical care the first time you sought it, in the last two weeks;
- Got medical care and prescriptions filled, in the last two weeks;
- Obtained all prescribed medicines (by some means);
- Obtained at least one of the medicines prescribed in the last health care visit;
- Obtained at least one of the medicines prescribed through the Brazilian Popular Pharmacy Program (Programa Farmácia Popular - PFP) in the last health care visit;
- Obtained at least one medicine through the public service, in the last health care visit;
- Have been hospitalized for 24 hours in the last 12 months; and
- Last hospitalization in a SUS unit in the last 12 months.

All questions used were dichotomized: no; yes.

The prevalence of the variables and respective 95% confidence intervals (95%CI) were calculated through stratification by sex (female; male), age groups (in years: 0 to 17; 18 to 29; 30 to 39; 40 to 59; 60 years and older), level of education/education of the head of the family (without schooling; incomplete elementary education; complete elementary education and incomplete high school; complete high school and incomplete higher education; complete higher education) and macro-region of the country (North; Northeast; Southeast; South; Midwest).

Data analysis was performed using the survey module of the Stata software version 16.1, which takes into account complex sampling design effects.

The project of the 2019 National Health Survey was submitted to the National Research Ethics Committee (*Comissão Nacional de Ética em Pesquisa* - CONEP)/National Health Council (*Conselho Nacional de Saúde* - CNS) and approved: Opinion No. 3,529,376, issued on August 23, 2019. All respondents were consulted, duly informed and agreed to take part in the survey.

## RESULTS

The estimated sample size was 108,457 households and 279,210 individuals. A total 293,725 individuals were investigated, of whom 51.9% were female, 24.1% were between 18 and 29 years of age, about 35.0% lived in the Northeast region and 36.7% had incomplete elementary education.

**Figure 1:**
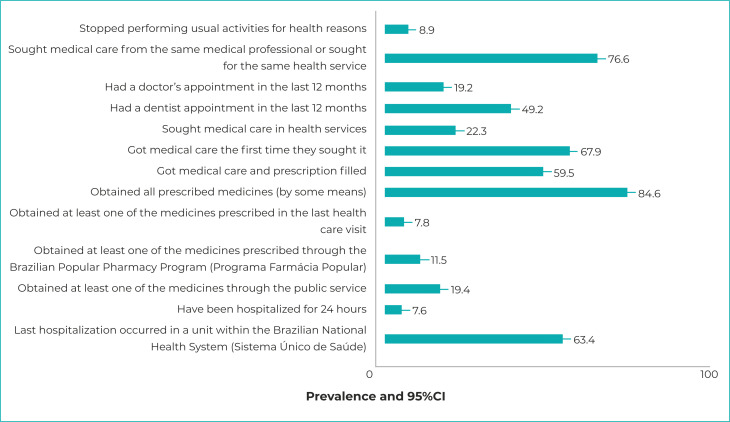
Prevalence of variables related to access and use of health services, National Health Survey, Brazil, 2019

Figure 1 shows the prevalence of the indicators investigated. It could be seen that most respondents (76.6%; 95%CI 75.9;77.2) used to seek the same place, doctor or health service, when they needed them, and 67.9% (95%CI 66.5;69.2) got medical care the first time they sought it; 19.2% (95%CI 18.5;19.9) had a doctor's appointment in the last 12 months and, in this same period, 49.2% of the respondents had a dentist appointment (95%CI 48.5;49.9). Regarding the use of medicines, 6 out of 10 users had their prescription filled in the last health care visit and of these, 84.6% (95%CI 83.1;86.0) were able to obtain all medicines; however, only 11.5% (95%CI 10.4;12.9), obtained at least one prescribed medicine through the Brazilian Popular Pharmacy Program, and 19.4% (95%CI 17.8;21.1) obtained medicines through a public service. As for hospital admission in the last year, 7.6% (95%CI 7.2;7.9) of the respondents used this service, and for 63.4% (95%CI 60.8;65.9) of them, the hospitalization was via the SUS.

When stratifying the sample by sex, it could be seen a higher proportion of females (11.6%; 95%CI 10.7;11.7) who stopped performing usual activities for health reasons, in the two weeks prior to the survey. Males showed a lower proportion of medical consultations in the last 12 months (66.6%; 95%CI 66.3;66.8), a lower number of dental visits (45.2%; 95%CI 44.3;46.2) and they also were less likely to seek health care in the two weeks prior to the study (17.6%; 95%CI 17.1;18.4). The other indicators presented similar proportions between the sexes, as shown in Table 1.

**Table 1:** Frequency of access to and utilization of health services according to sex, National Health Survey, Brazil, 2019

Variable	n	Sex			
		Female		Male	
		%	95%CI^a^	%	95%CI
Stopped performing usual activities for health reasons^b^	279,382	11.6	10.7;11.7	6.6	6.2;7.0
Sought medical care from the same medical professional or sought for the same health service	279,382	77.5	76.7;78.3	75.5	74.6;76.3
Had a doctor's appointment in the last 12 months	206,384	80.6	80.4;80.8	66.6	66.3;66.8
Had a dentist appointment in the last 12 months	279,382	52.8	51.9;53.7	45.2	44.3;46.2
Sought medical care in health services^b^	279,382	26.5	25.8;27.3	17.6	17.1;18.4
Got medical care the first time they sought it^b^	6,615	29.4	27.7;31.1	29.8	27.6;32.0
Got medical care and prescriptions filled^b^	40,357	59.8	58.0;61.6	58.9	56.6;61.2
Obtained all prescribed medicines (by some means)^c^	24,753	83.6	81.7;85.3	86.1	83.8;88.1
Obtained at least one of the medicines prescribed in the last health care visit^c^	24,753	8.4	7.1;9.9	6.9	5.5;8.6
Obtained at least one of the medicines prescribed through the Brazilian Popular Pharmacy Program in the last health care visit^c^	22,548	11.4	10.0;13.0	11.8	9.9;14.0
Obtained at least one medicine through the public service, in the last health care visit^c^	20,501	19.4	17.4;21.6	19.3	16.9;22.0
Have been hospitalized for 24 hours^d^	279,382	8.8	8.4;9.3	6.1	5.7;6.6
Last hospitalization occurred in a unit within the Brazilian National Health System	17,392	64.3	60.9;67.2	61.9	58.2;65.3

a) 95%CI: 95% confidence interval;

b) In the two weeks prior to the survey;

c) In the last health care visit;

d) In the 12 months prior to the survey.

Table 2 shows the distribution of indicators according to age groups. The results revealed a progressive increase in the prevalence of usual activities due to health reasons, as age increased, from 5.2% (95%CI 3.5;7.8) among individuals aged 0 to 17 years to 12.0% (95%CI 11.4;12.8) in older adults. The same occurred with indicators regarding seeking health services in the last two weeks, access to medicines through the Brazilian Popular Pharmacy Program, access to medicines through a public health service, a doctor's appointment in the last 12 months and hospitalization in the last 12 months. In turn, a dentist appointment in the last 12 months showed a lower prevalence with increasing age (Table 2).

**Table 2:** Frequency of access to and utilization of health services according to age groups, National Health Survey, Brazil, 2019

Variable	n	Age groups (in years)									
		0 to 17		18 to 29		30 to 39		40 to 59		60 years and older	
		%	95%CI^a^	%	95%CI^a^	%	95%CI^a^	%	95%CI^a^	%	95%CI^a^
Stopped performing usual activities for health reasons^b^	279,382	5.2	3.5;7.8	5.4	4.7;6.0	7.2	6.5;7.9	9.0	8.2;9.9	12.0	11.4;12.8
Sought medical care from the same medical professional or sought for the same health service	279,382	78.3	75.7;80.7	74.8	73.5;76.0	75.9	74.7;77.0	76.2	75.3;77.2	78.8	77.8;79.7
Had a doctor's appointment in the last 12 months	206,384	66.6	63.1;69.8	69.9	69.5;70.4	77.3	76.2;78.4	78.0	77.6;78.5	85.0	84.7;85.3
Had a dentist appointment in the last 12 months	279,382	59.3	55.7;62.9	55.1	53.6;56.6	56.1	54.8;57.3	50.8	49.6;51.7	35.2	33.9;36.5
Sought medical care in health services^b^	279,382	13.4	10.1;16.7	15.8	14.9;16.9	19.0	17.9;20.1	23.7	22.8;24.7	29.4	28.3;30.4
Got medical care the first time they sought it^b^	6,615	80.7	71.3;87.5	67.1	63.5;70.6	71.1	68.2;73.8	66.0	63.7;68.2	68.1	66.0;70.3
Got medical care and prescriptions filled^b^	40,357	59.7	46.8;71.5	58.2	54.1;62.3	58.0	54.5;61.3	60.0	57.5;62.6	60.0	57.6;62.4
Obtained all prescribed medicines (by some means)^c^	24,753	71.9	47.3; 87.9	86.9	83.5;89.6	83.7	79.3;87.3	84.2	82.2;86.0	85.5	83.1;87.5
Obtained at least one of the prescribed medicines^c^	24,753	9.3	2.5;29.2	7.1	5.2;9.6	9.1	4.9;10.1	8.1	6.8;9.8	6.5	5.2;8.2
Obtained at least one of the medicines prescribed through the Brazilian Popular Pharmacy Program^c^	22,548	4.6	0.9;19.2	5.6	3.4;9.2	6.3	4.3;9.0	11.0	9.2;13.2	17.7	15.4;20.4
Obtained at least one medicine through the public service^c^	20,501	10.5	4.1;24.3	16.6	12.2;22.1	15.2	11.6;19.7	17.8	15.5;20.3	25.2	22.1;28.4
Have been hospitalized for 24 hours^d^	279,382	4.7	3.1;7.0	6.0	5.4;6.7	7.3	6.6;8.0	7.0	6.5;7.5	10.4	9.8;11.1
Last hospitalization occurred in a unit within the Brazilian National Health System	17,392	82.1	63.5;92.4	68.4	61.7;73.6	56.3	50.4;62.0	62.5	58.5; 66.4	65.0	61.7;68.2

a) 95%CI: 95% confidence interval;

b) In the two weeks prior to the survey;

c) In the last health care visit;

d) In the 12 months prior to the survey. 12 months prior to the survey.

Table 3 shows the indicators according to the country's macro-regions. The North region presented the lowest proportion of people who had a doctor's and dentist appointments in the last 12 months, 69.1% (95%CI 68.8;70.0) and 41.3% (95%CI 39.9;42.8) respectively. The Northeast region had the highest proportion of last hospitalization via the SUS, 77.1% (95%CI 73.9;80.0), and greatest success in getting medical care the first time they sought it, 72.7% (95%CI 70.1;74.5). The highest proportion of obtaining at least one of the medicines prescribed in the last health care visit, through the Brazilian Popular Pharmacy Program, was observed in the South region: 17.8% (95%CI 15.1;21.0).

**Table 3:** Frequency of access to and utilization of health services according to the country's macro-regions, National Health Survey, Brazil, 2019

Variable	n	Macro-regions									
		North		Northeast		Midwest		Southeast		South	
		%	95%CI^a^	%	95%CI^a^	%	95%CI^a^	%	95%CI^a^	%	95%CI^a^
Stopped performing usual activities for health reasons^b^	279,382	9.0	8.4;9.7	9.6	9.1;10.2	8.7	7.9;9.6	9.0	8.4;9.6	8.1	7.4;8.8
Sought medical care from the same medical professional or sought for the same health service	279,382	70.5	67.9;71.0	76.0	74.9;77.0	71.0	69.0;72.3	78.8	77.7;80.0	77.3	75.7;78.8
Had a doctor's appointment in the last 12 months	206,384	69.1	68.8;70.0	71.6	71.3;71.8	74.4	73.9;74.9	80.5	80.2;80.8	78.0	77.6;78.5
Had a dentist appointment in the last 12 months	279,382	41.3	39.9;42.8	42.4	41.4;43.4	48.4	46.8;50.1	53.0	51.8;54.3	54.0	52.5;55.5
Sought medical care in health services^b^	279,382	16.6	15.7;17.6	19.5	18.8;20.3	19.7	18.5;20.9	25.1	24.1;26.2	23.5	22.4;24.6
Got medical care the first time they sought it^b^	6,615	63.2	60.0;66.3	72.7	70.1;74.5	70.5	67.5;73.3	68.5	66.0;70.9	59.9	56.9;62.7
Got medical care and prescriptions filled^b^	40,357	62.2	58.9;65.4	57.6	55.4;59.7	61.8	58.4;65.0	59.2	56.6;61.9	61.1	57.9;64.3
Obtained all prescribed medicines (by some means)^c^	24,753	81.5	78.4;84.3	83.7	81.4;85.7	85.1	82.0;87.7	84.5	82.3;87.3	85.3	82.3;87.9
Obtained at least one of the prescribed medicines^c^	24,753	8.9	7.1;11.1	8.0	6.5;9.9	5.7	4.2;7.6	8.0	6.3;10.0	7.8	6.1;9.9
Obtained at least one of the medicines prescribed through the Brazilian Popular Pharmacy Program^c^	22,548	8.7	6.6;11.5	7.8	6.5;9.4	12.1	9.4;15.4	11.5	9.5;13.8	17.8	15.1;21.0
Obtained at least one medicine through the public service^c^	20,501	19.4	16.0;23.3	17.5	15.2;20.2	15.2	12.3;18.7	20.3	17.3;23.7	18.9	15.9;22.3
Have been hospitalized for 24 hours^d^	279,382	6.6	6.0;7.2	6.7	6.2;7.1	9.2	8.5;10.1	7.9	7.2;8.5	8.1	7.4;8.8
Last hospitalization occurred in a unit within the Brazilian National Health System	17,392	73.1	68.3;77.4	77.1	73.9;80.0	60.8	56.1;65.4	55.6	50.8;60.4	63.1	58.6;67.4

a) 95%CI: 95% confidence interval;

b) In the two weeks prior to the survey;

c) In the last health care visit;

d) In the 12 months prior to the survey.

As for schooling, an inversely proportional relationship was found, with a higher prevalence among those with low level of education, when taking into consideration the following indicators: stopped performing usual activities for health reasons in the two weeks prior to the survey (14.0%; 95%CI 12.7;15.4); had a health care visit in the last two weeks and obtained prescribed medication (65.8%; 95%CI 61,3;70,0); obtained, through the Brazilian Popular Pharmacy Program, at least one of the medicines prescribed in the last health care visit (16.5%; 95%CI 12.2;22.0); and obtained, through the public health service, at least one of the medicines prescribed in the last health care visit (13.2%; 95%CI 9.0;18.9). However, the participants with high level of education showed a higher proportion of dental visits in the last 12 months (71.9%; 95%CI 70.6;73.2) and also a higher proportion of obtaining all the medicines prescribed in the last health care visit (90.0%; 95%CI 87.0;92.4), when compared to participants without schooling or low level of education (Table 4).

**Table 4:** Frequency of access to and utilization of health services according to level of education, National Health Survey, Brazil, 2019

Variable	n	Level of education									
		Without schooling		Incomplete elementary education		Complete elementary education/Incomplete high school		Complete high school/Incomplete higher education		Complete higher education	
		%	95%CI^a^	%	95%CI^a^	%	95%CI^a^	%	95%CI^a^	%	95%CI^a^
Stopped performing usual activities for health reasons^b^	279,382	14,0	12,7;15,4	11,3	10,6;12,0	7,6	4,0;8,5	7,3	6,8;7,9	7,5	6,7;8,3
Sought medical care from the same medical professional or sought for the same health service	279,382	78,5	76,8;80,2	79,2	78,3;80,0	76,0	74,6;77,3	74,4	73,3;75,4	75,9	74,6;77,2
Had a doctor's appointment in the last 12 months	206,384	75,9	75,4;76,4	79,4	78,5;80,3	69,6	69,1;70,3	66,5	65,8;67,1	88,0	87,1,88,9
Had a dentist appointment in the last 12 months	279,382	20,3	18,8;21,9	35,0	33,9;36,1	50,1	48,5;51,6	56,6	55,5;57,6	71,9	70,6;73,2
Sought medical care in health services^b^	279,382	23,3	21,6;25,1	23,6	22,6;24,5	19,7	18,5;21,0	20,6	19,7;21,6	26,2	24,9;27,6
Got medical care the first time they sought it^b^	6,615	21,9	18,9;25,2	29,1	26,9;31,4	30,7	27,0;34,6	30,7	28,5;33,1	30,0	27,3;32,8
Got medical care and prescriptions filled^b^	40,357	65,8	61,3;70,0	61,9	59,2;64,5	62,2	58,4;65,9	59,2	56,6;61,8	51,4	48,0;54,8
Obtained all prescribed medicines (by some means)^c^	24,753	79,7	73,9;84,4	84,3	82,2;86,2	81,5	76,5;85,7	84,6	81,8;87,1	90,0	87,0;92,4
Obtained at least one of the prescribed medicines^c^	24,753	13,2	9,0;18,9	8,2	6,8;10,0	8,2	6,0;11,2	7,3	5,5;9,7	5,1	3,5;7,4
Obtained at least one of the medicines prescribed through the Brazilian Popular Pharmacy Program^c^	22,548	16,5	12,2;22,0	14,2	12,1;16,6	13,3	10,2;17,0	8,7	6,9;10,9	7,6	5,4;10,6
Obtained at least one medicine through the public service^c^	20,501	30,4	24,6;37,0	24,4	21,4;27,5	25,8	20,8;31,5	13,8	11,4;16,7	9,5	7,0;12,8
Have been hospitalized for 24 hours^d^	279,382	10,9	9,7;12,3	8,1	7,6;8,8	6,5	5,8;7,3	7,0	6,5;7,6	7,5	6,7;8,4
Last hospitalization occurred in a unit within the Brazilian National Health System	17,392	90,1	86,2;92,9	79,4	75,7;82,6	75,2	69,5;80,1	55,0	50,2;59,5	20,7	16,2;26,1

a) 95%CI: 95% confidence interval;

b) In the two weeks prior to the survey;

c) In the last health care visit;

d) In the 12 months prior to the survey.

## DISCUSSION

The analysis of the main results of this study suggests inequalities in the access to and utilization of health services in Brazil. The findings showed a lower use of dental services among older adults, and for those who lived in the North and Northeast regions and among those with low level of education, additionally, a lower proportion of health care visits aimed at seeking for medicines through the Brazilian Popular Pharmacy Program or public service, highlighting the differences in this access among sociodemographic groups.

When comparing the results presented with data from the 2013 PNS, a small increase in access to medical and dental consultations was identified in the last 12 months, as well as in seeking care and the care provided in the last two weeks prior to the survey, a result also identified in other national studies.^5,12^ Paradoxically, the proportional reduction of those who got medical care the first time they sought it, from 95.3% in 2013 to 67.9% in 2019, is noteworthy.^5^ This result represents a setback in the advances that the Brazilian National Health System has achieved over the last 30 years, especially in primary health care, a possible reflection of the government initiatives that have been adopted since 2017, and caused a drastic reduction in funding, decrease in health services and human resources, leading to the weakening of services and health care for the SUS users.^6,12^

In this sense, access to medicines through the Brazilian Popular Pharmacy Program and public services was reduced by about 15%, between 2013 and 2019. For 15 years, PFP has been expanding and promoting the population's access to medicines, especially among older adults and people with chronic non-communicable diseases (NCDs), therefore it received recognition from the World Health Organization (WHO), given the results achieved.^13,14^ Moreover, the reduction in access to medicines mainly exposes older adults and chronically ill people, who are the biggest users of these programs, a situation that is likely to worsen as the demographic and epidemiological transition advances.^15,16^

Regarding sex, men have presented a lower access and use of health services, a fact that is widely discussed in the national and international literature.^17–19^ Men, in addition to presenting a higher prevalence of health risk behaviors, such as smoking, alcohol consumption and sedentary lifestyle, are also less likely to seek health care, which increases the chances of long-term problems.^17^ Accordingly, Bibiano et al. showed, in their study, that the care of elderly men has a curative and rehabilitation profile, rather than prevention and health promotion.

The highest frequency of seeking medical care and the lowest immediate access to dental care were mostly identified among older adults. The physiological process of aging, together with the highest occurrence of chronic diseases in this population, in addition to the promotion of public policies aimed at increasing and facilitating access to services, makes older adults seek more health care, a demand that has been increasing in recent years, with an upward perspective, even though the difficulty of access in some areas reinforces an important gap in the supply of health services.^4,20^

Furthermore, regarding the macro-region of residence, the results of this study portray the regional inequalities in the access and use of health services in Brazil, a fact recognized by the Ministry of Health through initiatives such as the establishment of the Brazilian National Primary Health Care Policy (*Política Nacional de Atenção Básica* – PNAB) and conduction of other population-based surveys, such as the Chronic Disease Risk and Protective Factors Surveillance Telephone Survey (*Sistema de Vigilância de Fatores de Risco e Proteção para Doenças Crônicas por Inquérito Telefônico* - VIGITEL).^21,22^ The lowest access to medical and dental consultation in the North and Northeast regions is associated with the lowest availability of these professionals in the public network, because, although in recent years, policies to expand health services in Brazil have focused on the most vulnerable regions and reduced inequalities, the guarantee of human resources to provide care for the population is still difficult.^6,21,23^

Stratification by level of education reinforces inequalities in access to health services. Although trend studies have shown increased access to dental care in Brazil,^12^ barriers such as low income, low level of education and poor supply of public oral health services are still obstacles to meet the existing demands, especially for older adults.^24,25^ The results of this study corroborate the literature by showing that the availability of oral health care is still limited for the most vulnerable people. It is worth mentioning that 70% of the Brazilian population relies on the SUS as their only source of access to health services, in addition to the fact that the number of dental services distributed over the national territory is insufficient to provide care for the general population, causing repressed demand and difficulty of access to services.^26^

Socioeconomic status also appears as a factor related to access to and utilization of different health services: higher income or better socioeconomic status maintains a direct relationship with a higher probability of using health services, such as access to and use of medicines.^27–29^ Thus, it can be inferred that those with lower income or lower socioeconomic status have greater difficulty and dependence on the SUS in order to access to medicines.^30^ The study also showed that people with low level of education obtained, through the Brazilian Popular Pharmacy Program, at least one of the medicines prescribed in the last health care visit, and they obtained through the public health service at least one of the medicines prescribed in the last health care visit. These results reinforce the principle of equity promoted by the SUS in access to medicines, overcoming disparities between social groups within this health service.

This study presents as a limitation the method used to obtain the information from the PNS, extrapolated here for the population, although it was provided by a single individual, as representative of the other household members, in addition to adopting the level of education in the stratification of the population by income and not taking into consideration the per capita income. However, the study was able to analyze important health characteristics of the Brazilian population, by taking into account that, despite the improvement observed in some indicators, such as a higher number of medical and dental consultations, the SUS – as the main and often, the only way to access health services for the low-income population – is still insufficient, since great regional inequality persists: the North and Northeast regions have the least access to services, and individuals with low socioeconomic status are those who face more difficulties in having their demands met.

The results presented indicate the relevance of monitoring the sociodemographic characteristics and vulnerabilities of the population. Knowledge of these conditions allows us to understand their influence and thus contribute to a more effective performance of the Brazilian National Health System in order to guide equitable health policies, aimed at identifying risks and population demands for access to and utilization of health services in Brazil.
